# Atg16L1 as a Novel Biomarker and Autophagy Gene for Diabetic Retinopathy

**DOI:** 10.1155/2021/5398645

**Published:** 2021-03-18

**Authors:** Xinxiao Gao, Yunhui Du, Wayne Bond Lau, Yu Li, Siquan Zhu, Xin-Liang Ma

**Affiliations:** ^1^Department of Physiology and Pathophysiology, School of Basic Medical Sciences, Capital Medical University, Beijing 100069, China; ^2^Department of Ophthalmology, Beijing Anzhen Hospital, Capital Medical University, Beijing 100029, China; ^3^Beijing Anzhen Hospital, Capital Medical University, Beijing Institute of Heart, Lung and Blood Vessel Diseases, Beijing 100029, China; ^4^Department of Emergency Medicine, Thomas Jefferson University, 1025 Walnut Street, College Building, Suite 808, Philadelphia, PA 19107, USA

## Abstract

**Objective:**

Accumulating evidence suggests the critical role of autophagy in the pathogenesis of diabetic retinopathy (DR). In the current study, we aim to identify autophagy genes involved in DR via microarray analyses.

**Methods:**

Gene microarrays were performed to identify differentially expressed lncRNAs/mRNAs between normal and DR retinas. Gene Ontology and Kyoto Encyclopedia of Genes and Genomes analyses of lncRNA-coexpressed mRNAs were used to determine the related pathological pathways and biological modules. Real-time polymerase chain reactions (PCR) were conducted to validate the microarray analyses.

**Results:**

A total of 2474 significantly dysregulated lncRNAs and 959 differentially expressed mRNAs were identified in the retina of DR. Based upon Signalnet analysis, Bcl2, Gabarapl2, Atg4c, and Atg16L1 participated the process of cell death in DR. Moreover, real-time PCR revealed significant upregulation of Atg16L1.

**Conclusion:**

This study indicated the importance and potential role of Atg16L1, one of the autophagy genes, as a biomarker in DR development and progression.

## 1. Introduction

Diabetic retinopathy (DR) is a major contributor to vision loss in patients with diabetes mellitus [1]. DR incidence has been increasing rapidly in recent years, from 127 million in 2010 to a projected 191 million by 2030 [2]. The underlying key biochemical pathways may include genetic and epigenetic factors, polyol pathway activation, production of advanced glycation endproducts (AGEs), protein kinase C (PKC) activation, hexosamine pathway activation, and poly (ADP-ribose) polymerase upregulation. However, DR pathogenesis is complex and remains incompletely understood [3].

Autophagy is the primary intracellular catabolic mechanism mediating degradation and recycling of proteins and organelles. Due to its essential role in development, aging, starvation, cellular differentiation, and cell death, autophagy has attracted marked attention in recent years. Dysregulation of autophagy and lysosomal pathways is the hallmark of many diseases, from diabetes to neurodegenerative disorders and lysosomal storage diseases [4–6]. Moreover, growing data have suggested the crucial role of autophagy in DR pathophysiology [7, 8]. Heretofore, there remains limited understanding regarding the exact autophagy genes involved in DR development and progression. The advent of microarray technology has facilitated detection of the comprehensive pattern of simultaneous transcript expression [9]. In this study, we aim to identify the autophagy genes involved in DR by microarray analyses.

## 2. Methods

### 2.1. Diabetic Mouse Model

All animal procedures were approved in accordance with the Association of Research in Vision and Ophthalmology Treatment of Animals in Research and the Capital Medical University's Animal Care and Use Committee Guidelines. All in vivo experiments were performed upon adult male C57BL/6 mice (8 weeks old). Experimental mice were randomized to receive HFD (60 kcal%) (Research Diets Inc. D12492i) or normal diet control (ND, D12450Bi). Eight months after ND or HFD, mice were anesthetized with 2% isoflurane. Blood glucose concentrations were measured 48 hours after STZ injection and weekly thereafter. Only animals with blood glucose levels exceeding 250 mg/dL were considered diabetic. After 10–12 weeks, the animals were sacrificed by pentobarbital overdose. The retinas were quickly removed, placed in liquid nitrogen, and stored at −80°C for biochemical measurement.

### 2.2. Microarray Analysis

Total RNAs were isolated from the retinas of diabetic mice and age-matched controls using TRIzol reagent (Life Technologies, Carlsbad, CA, USA) and purified with an RNeasy mini kit (Qiagen, Valencia, CA, USA) per manufacturer's protocol. Microarray profiling was performed by mouse Clariom™ D Assay (Affymetrix GeneChip®, USA, an assay containing 65956 gene-level probe sets). Raw data were normalized at the transcript level by the TAC software (Transcriptome Analysis Console; version: 4.0.1) using Affymetrix default analysis settings (Robust Multichip Analysis workflow). The median summarization of transcript expressions was calculated.

Based upon differentially expressed lncRNAs, hierarchical clustering was performed by R package heatmap (version: 1.0.12). Gene Ontology (GO) analysis determined the main function of the differentially expressed genes, yielding the likely gene regulatory network on the basis of biological processes and molecular function. Specifically, a two-sided Fisher's exact test and chi-square test were used to classify the GO category. The false discovery rate (FDR) was calculated to correct the *P* value (the smaller the FDR, the small the error in judging the *P* value). Pathway analysis was conducted upon the differential genes identified, per Kyoto Encyclopedia of Genes and Genomes (KEGG) databases. Fisher's exact test was performed to select the significant pathway. The threshold of significance was considered *P* < 0.05. Based the interactions of genes in the KEGG database, global signal transduction network (Signalnet) was generated to demonstrate the interaction between the differentially expressed genes in treated groups. The visualization of network was built by software Cytoscape (version: 3.6.0).

### 2.3. Real-Time Quantitative PCR

Quantitative real-time PCR (qRT-PCR) was applied to validate the selected genes from the microarray analyses. Total RNAs were extracted using TRIzol reagent (Invitrogen, Carlsbad, Canada) and reverse-transcribed per manufacturer's instructions. qRT-PCR reaction was monitored by the ABI Prism 7500 Sequence Detection System (Applied Biosystems, Foster City, CA) and run in duplicate for each sample. The PCR reaction mixture (20 *μ*l) contained 2 *μ*l of cDNA template, 0.6 *μ*l forward and reverse primers, and 10 *μ*l of 2×SYBR-Green PCR Mix (Takara). The level of mRNA expression was calculated from the fluorescence intensity (b-actin served as internal control). Primers targeting Atg4c (F: 5′-GATGAAAGCAAGATGTTGCCTG-3′ and R: 5′-TCTTCCCTGTAGGTCAGCCAT-3′) and Atg16L1 (F: 5′-CAGAGCAGCTACTAAGCGACT-3′ and R: 5′-AAAAGGGGAGATTCGGACAGA-3′) were used for real-time RT-PCR amplification. The relative gene expression was calculated by the *ΔΔ* threshold cycle (Ct) method. Real-time PCR reaction was run in biological triplicates for each sample. Melting curve analysis was used to verify the product purity at the end of the PCR run.

## 3. Results

### 3.1. Overview of lncRNA-mRNA Microarray Analysis

**Figure 1:**
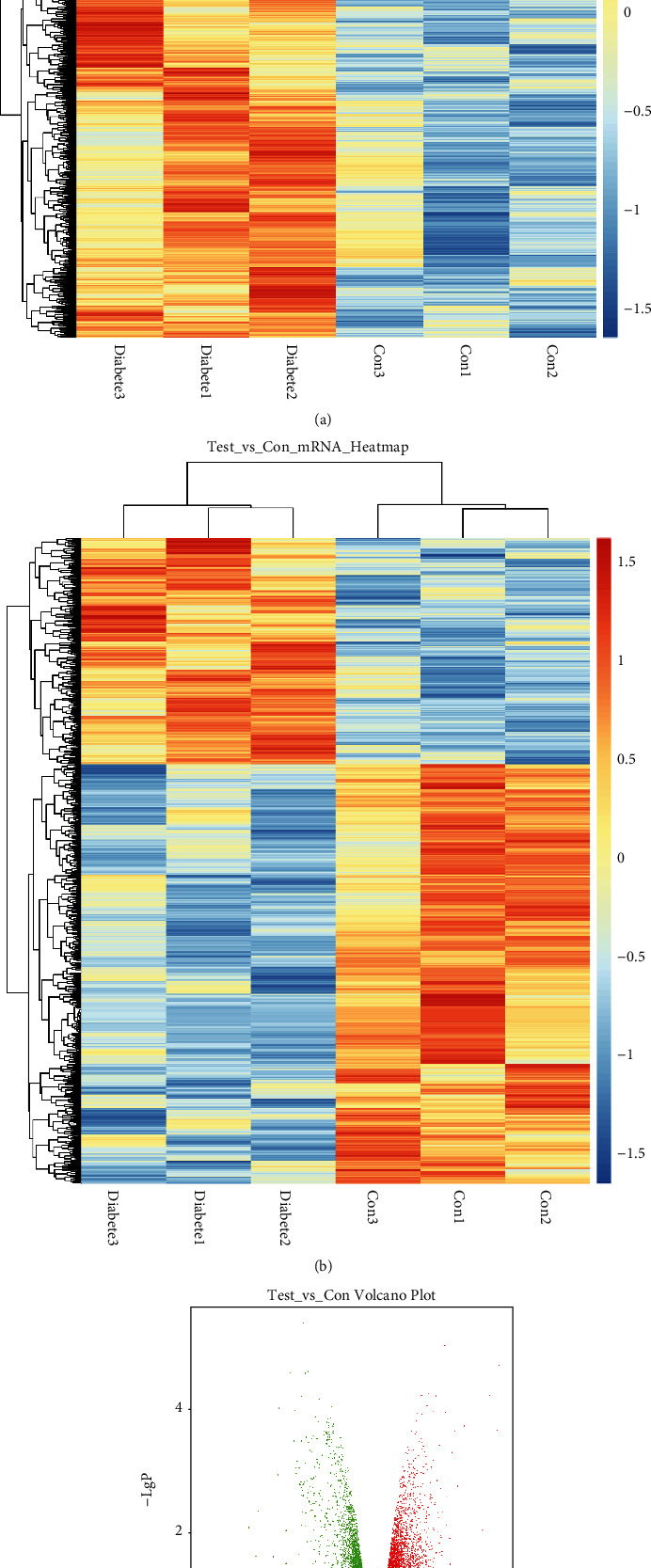
Identification of DR-related lncRNAs/mRNAs by microarray analysis. (a) Heatmap from the hierarchical clustering analysis demonstrating the differentially expressed lncRNAs between nondiabetic and diabetic retinas. (b) Heatmap demonstrating the dysregulated mRNAs between nondiabetic and diabetic retinas. (c) Volcano plot illustrating the differentially expressed lncRNAs in nondiabetic and diabetic retinas.

To reveal a differential gene expression profile, hierarchical clustering analysis compared lncRNA-mRNA expression between diabetic and nondiabetic retinas (Figures 1(a) and 1(b)). Differentially expressed lncRNAs (with statistical significance) between the two groups were identified via volcano plot filtering (Figure 1(c)). The combined criteria of a *P* value <0.05 and fold change > 1.1 identified 2474 lncRNAs expressed differentially, including 1487 upregulated and 987 downregulated lncRNAs. 317 significantly increased and 642 decreased mRNAs were also identified. The top 10 differentially expressed lncRNAs and mRNAs between diabetic and nondiabetic retinas are listed in Tables 1 and 2, respectively.

### 3.2. Gene Enrichment and Pathway Analysis of lncRNA-Coexpressed mRNAs

**Figure 2:**
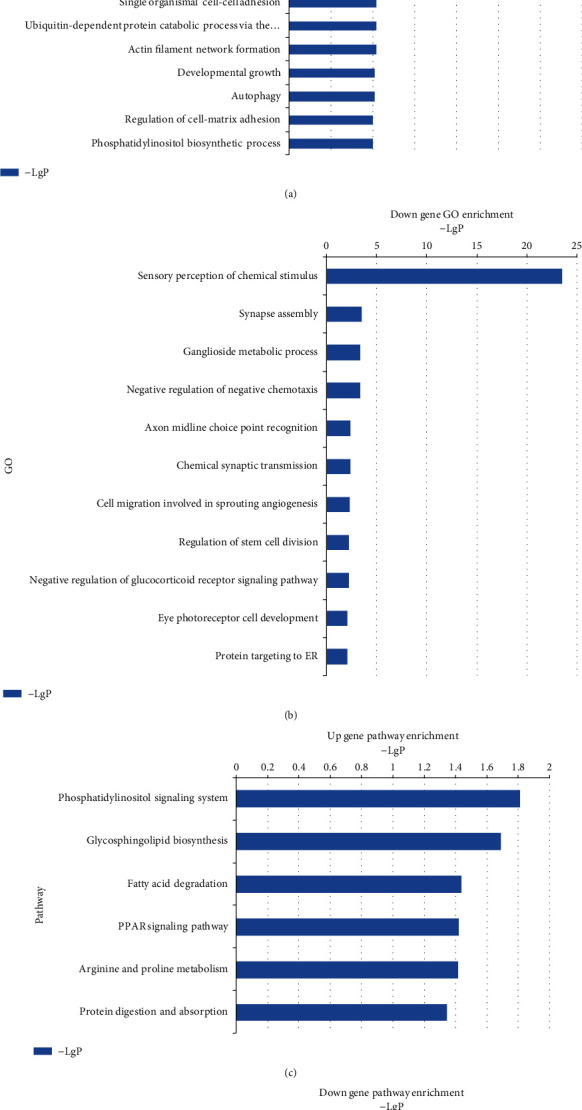
Gene Ontology (GO) analyses and KEGG analysis of the dysregulated lncRNAs with top enrichment scores of biological processes. (a) Significantly upregulated differentially expressed genes, by GO analysis. (b) Significantly downregulated differentially expressed genes, by GO analysis. (c) Significant pathways of differentially expressed upregulated genes. (d) Significant pathways of differentially expressed downregulated genes.

GO analysis determined the main gene functions and gene product enrichment affected by diabetes. The GO database revealed (of 21 upregulated GOs) photoreceptor cell morphogenesis, activation of MAPK activity, and autophagy were related to retinal function (Figure 2(a)). Of 11 downregulated GOs, only eye photoreceptor cell development was directly related to retinal function (Figure 2(b)). The summaries of those genes involved in the significant signaling pathways relevant to retinal function are listed in Table 3. KEGG analysis revealed highly enriched upregulated signaling pathways included phosphatidylinositol signaling system, glycosphingolipid biosynthesis, fatty acid degradation, PPAR signaling pathway, arginine and proline metabolism, and protein digestion and absorption (Figure 2(c)). Significantly downregulated pathways included biosynthesis of unsaturated fatty acids, protein export, nitrogen metabolism, and calcium signaling pathway (Figure 2(d)).

### 3.3. Construction of the lncRNA-mRNA Coexpression Network and Identification of Genes in the Process of Cell Death

**Figure 3:**
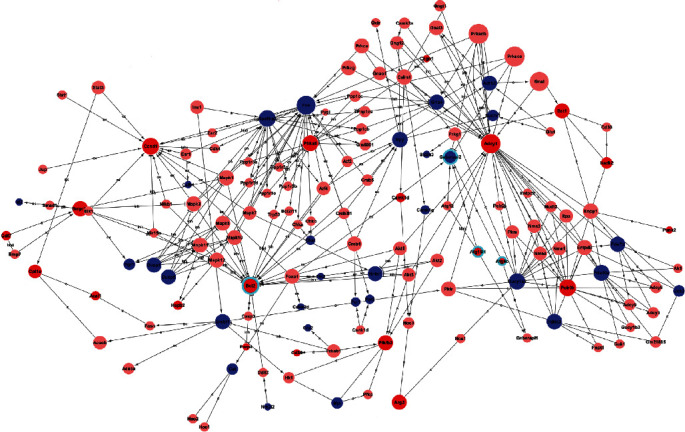
Signalnet of cell death-related lncRNA-mRNA coexpression network (the circles highlighted with green ring). The red circles represent upregulated genes, and blue circles represent downregulated genes. Interaction between the genes is shown as follows: a: activation; b: binding/association; c: compound; ex: expression; inh: inhibition; ph: phosphorylation; ubi: ubiquination.

**Figure 4:**
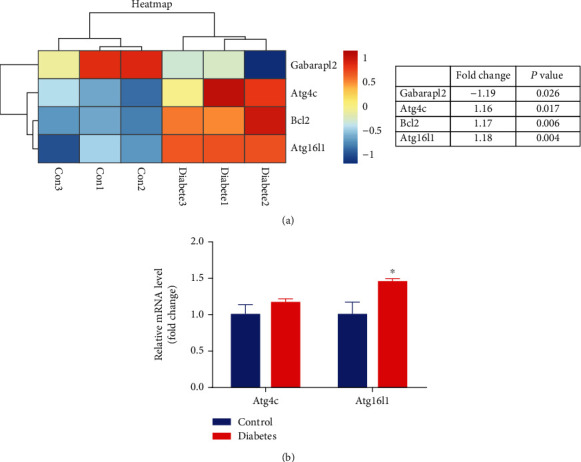
Differential expression of cell death-related genes between nondiabetic and diabetic retinas. (a) Heatmap from microarray analysis of gene expression related to the process of cell death. (b) qRT-PCR analysis was performed on the two genes involved in the process of autophagy. The results showed that, compared to control, Atg16L1 was significantly upregulated while Atg4c expression was unchanged in diabetic retinas.

Based upon significant pathway and GO analysis, the Signalnet analysis screened the key genes associated with DR pathogenesis. A total of 369 key genes were identified in the transduction network. Specifically, Bcl2, Gabarapl2, Atg4c, and Atg16L1 were involved in the process of cell death.  Figure 3 showed the Signalnet of cell death-related lncRNA-mRNA coexpression network. To evaluate the expression of these 4 specific genes in diabetic and nondiabetic retinas, hierarchical clustering analysis was further performed. The heatmap (Figure 4(a)) suggested the good classification of mRNA expression profile between those two groups.

### 3.4. Confirmation of Gene Expression in Autophagy by RT-PCR

To further validate the microarray analysis results, we conducted qPCR assays for the genes involved in autophagy. qRT-PCR analysis revealed significantly upregulated expression of Atg16L1 compared to control, while Atg4c expression was unremarkable (Figure 4(b)). The potential role of Atg16L1 in DR pathogenesis was further supported.

## 4. Discussion

The pathogenesis of DR is hugely complex and likely implicates the dysregulation of many biochemical and molecular signaling pathways. Despite great strides, the detailed molecular mechanisms responsible for DR are incompletely known. Recent studies have demonstrated that autophagy participates in DR pathology [10, 11], but the underlying responsible genes are unclear. To better understand the significance of autophagy in DR, we evaluated the associated genes by microarray analyses. Signalnet analysis suggests the involvement of Bcl2, Gabarapl2, Atg4c, and Atg16L1 in the process of cell death in DR. As expression of Atg16L1 was significantly increased, this particular molecule may be an important participant in DR development and progression.

lncRNAs/mRNAs have garnered attention in recent years for their potential regulatory role in DR [12, 13]. Additionally, the important roles of lncRNAs were investigated in PDR by harvesting fibrovascular membranes [14]. In the current study, we identified 2474 significantly dysregulated lncRNAs and 959 differentially expressed mRNAs, further confirmed by PCR analysis. In another prior study, lncRNAs were investigated with respect to DR pathogenesis in a mouse streptozotocin-induced diabetic model, utilizing microarray analyses. Only 303 aberrantly expressed lncRNAs were identified in the retinas of early DR [13]. Different animal models and protocol may lead to this discrepancy. Future bioinformatic analysis of the lncRNAs/mRNAs will be helpful to confirm these results.

Accumulating evidence suggests that autophagy plays an essential role and may act as a double-edged sword in DR [15, 16]. Activation of autophagy results in cell survival under mild stress in DR, while dysregulated autophagy can lead to massive cell death during severe stress conditions. In high glucose concentrations, autophagy activation in cultured ARPE-19 cells decreases proinflammatory cytokine production [17]. High glucose upregulates autophagy in retinal Müller cells during early DR pathogenesis phases [10]. In agreement with these previous studies, we demonstrate (based on GO analysis) that autophagy is upregulated during DR. While the contribution of autophagy to DR development requires further study, our findings may open interesting perspectives for novel therapies.

Interestingly, two conserved mRNAs, including Atg16L1, were identified to be involved with autophagy in the current study. As a key player in early autophagy initiation, Atg16L1 also regulates subsequent steps of this pathway [18]. Moreover, recent progress has demonstrated that Atg16L1 may be involved in diabetic pathophysiology by regulating autophagy [19, 20]. In consistent fashion, we report significantly increased expression of Atg16L1 in diabetic mouse retinas. Based upon these findings, we hypothesize that Atg16L1 may be an important component of the DR pathological process. Further studies are warranted to better understand the functional role of this novel mRNA in DR.

In conclusion, we identified dysregulated autophagy genes involved in DR by microarray analyses. Atg16L1 may be a potential biomarker for the diagnosis and prognosis of DR. It may serve as a potential therapeutic target blocking and slowing DR progression. Future work is required to confirm these findings and elucidate the specific underlying molecular signaling mechanisms.

## Figures and Tables

**Table 1:** Top ten differentially expressed lncRNAs in diabetic retinas compared to undiabetic retinas.

lncRNAs	Strand	*P* value	Fold change
*Upregulated*			
Chr18: 65390334-65393029	Forward	0.0002	2.63
Chr10: 122606616-122609483	Reverse	0.0005	1.84
Chr15: 73979542-73994040	Forward	0.0007	1.58
Chr7: 35838522-35839628	Forward	0.0008	1.56
Chr4: 129830892-129833771	Forward	0.0015	1.55
Chr5: 43784046-43786117	Reverse	0.0017	1.44
Chr12: 81308672-81311207	Reverse	0.0018	1.37
Chr6: 84883190-84884193	Reverse	0.0025	1.35
Chr1: 132943512-132945320	Reverse	0.0028	1.33
Chr2: 129082743-129084444	Forward	0.0034	1.25
*Downregulated*			
Chr4: 10136920-10138026	Reverse	0.0001	1.60
Chr11: 3193516-3194263	Reverse	0.0008	1.55
Chr13: 51168717-51170674	Reverse	0.0020	1.38
Chr4: 3089552-3091186	Forward	0.0022	1.38
Chr1: 84984529-84984611	Forward	0.0026	1.36
Chr14: 42037609-42040012	Reverse	0.0028	1.29
Chr5: 131041336-131044079	Reverse	0.0028	1.27
Chr8: 73353725-73362159	Forward	0.0041	1.25
Chr10: 61858666-61859663	Reverse	0.0043	1.23
Chr7: 21523277-21524200	Reverse	0.0045	1.20

**Table 2:** Top ten differentially expressed mRNAs in diabetic retinas compared to undiabetic retinas.

Gene symbol	Description	*P* value	Fold change
*Upregulated*			
Hdc	Histidine decarboxylase	0.0004	1.66
Dnah7b	Dynein, axonemal, heavy chain 7B	0.0009	1.47
Erap1	Endoplasmic reticulum aminopeptidase 1	0.0010	1.47
Alpk2	Alpha-kinase 2	0.0011	1.36
Gsc2	Goosecoid homebox 2	0.0016	1.36
N4bp2l1	NEDD4 binding protein 2-like 1	0.0027	1.27
Skap2	src family-associated phosphoprotein 2	0.0028	1.25
Zscan29	Zinc finger SCAN domains 29	0.0033	1.25
Tmprss7	Transmembrane serine protease 7	0.0039	1.24
Atg16l1	Autophagy-related 16-like 1	0.0043	1.18
*Downregulated*			
Camk1g	Calcium/calmodulin-dependent protein kinase I gamma	0.0002	1.44
Vmn1r121	Vomeronasal 1 receptor 121	0.0003	1.43
Sly	Sycp3 like Y-linked	0.0003	1.40
Serinc5	Serine incorporator 5	0.0004	1.33
Clybl	Citrate lyase beta like	0.0018	1.32
Hist1h1c	Histone cluster 1, H1c	0.0018	1.31
Ubtd2	Ubiquitin domain containing 2	0.0022	1.27
Ttc12	Tetratricopeptide repeat domain 12	0.0030	1.26
Rhobtb1	Rho-related BTB domain containing 1	0.0036	1.26
Duxbl2	Double homeobox B-like 2	0.0037	1.25

**Table 3:** The involved genes in signaling pathways relevant to retinal function.

Pathway name	*P* value	Gene symbol
Photoreceptor cell morphogenesis	0.000933385	Cabp4, Grk1
Activation of MAPK activity	0.006855100	Map3k13, Fgf2, Adam9
Autophagy	0.009045081	Atg4c, Atg16L1

## Data Availability

The data used to support the findings of this study are available from the corresponding authors upon request.
